# Rapid Screening of Chemical Components in *Salvia miltiorrhiza* with the Potential to Inhibit Skin Inflammation

**DOI:** 10.3390/ijms25137369

**Published:** 2024-07-05

**Authors:** Kehang He, Yikao Hu, Xiaolin Bai, Xun Liao

**Affiliations:** 1 Chengdu Institute of Biology, Chinese Academy of Sciences, Chengdu 610041, China; hekh@cib.ac.cn (K.H.); huyk@cib.ac.cn (Y.H.); baixl@cib.ac.cn (X.B.); 2 University of Chinese Academy of Sciences, Beijing 100049, China

**Keywords:** *Salvia miltiorrhiza* roots, anti-inflammatory, hyaluronidase inhibitors, synergistic effects

## Abstract

Hyaluronidase possesses the capacity to degrade high-molecular-weight hyaluronic acid into smaller fragments, subsequently initiating a cascade of inflammatory responses and activating dendritic cells. In cases of bacterial infections, substantial quantities of HAase are generated, potentially leading to severe conditions such as cellulitis. Inhibiting hyaluronidase activity may offer anti-inflammatory benefits. *Salvia miltiorrhiza* Bunge, a traditional Chinese medicine, has anti-inflammatory properties. However, its effects on skin inflammation are not well understood. This study screened and evaluated the active components of *S. miltiorrhiza* that inhibit skin inflammation, using ligand fishing, enzyme activity assays, drug combination analysis, and molecular docking. By combining magnetic nanomaterials with hyaluronidase functional groups, we immobilized hyaluronidase on magnetic nanomaterials for the first time in the literature. We then utilized an immobilized enzyme to specifically adsorb the ligand; two ligands were identified as salvianolic acid B and rosmarinic acid by HPLC analysis after desorption of the dangling ligands, to complete the rapid screening of potential anti-inflammatory active ingredients in *S. miltiorrhiza* roots. The median-effect equation and combination index results indicated that their synergistic inhibition of hyaluronidase at a fixed 3:2 ratio was enhanced with increasing concentrations. Kinetic studies revealed that they acted as mixed-type inhibitors of hyaluronidase. Salvianolic acid B had K*_i_* and K*_is_* values of 0.22 and 0.96 μM, respectively, while rosmarinic acid had values of 0.54 and 4.60 μM. Molecular docking revealed that salvianolic acid B had a higher affinity for hyaluronidase than rosmarinic acid. In addition, we observed that a 3:2 combination of SAB and RA significantly decreased the secretion of TNF-α, IL-1, and IL-6 inflammatory cytokines in UVB-irradiated HaCaT cells. These findings identify salvianolic acid B and rosmarinic acid as key components with the potential to inhibit skin inflammation, as found in *S. miltiorrhiza*. This research is significant for developing skin inflammation treatments. It demonstrates the effectiveness and broad applicability of the magnetic nanoparticle-based ligand fishing approach for screening enzyme inhibitors derived from herbal extracts.

## 1. Introduction

Hyaluronic acid (HA) is a macromolecular polymer composed of *D*-glucuronic acid (GlcA) and *D*-*N*-acetylglucosamine (GlcNAc) units. Among these, GlcA and GlcNAc are linked by *β*-1,3-glycosyl bonds, while the disaccharide units are linked by *β*-1,4-glycosyl bonds [1,2]. HA serves several physiological functions, including maintaining cell structure, providing energy, regulating proteins, aiding in water and electrolyte diffusion and operation, lubricating joints, regulating blood vessel wall permeability, inhibiting inflammatory responses, etc. [3]. Hyaluronidase (HAase) is the enzyme that breaks down hyaluronic acid into smaller molecules [4]. Hyaluronidase is able to break down high-molecular-weight hyaluronic acid into smaller pieces, triggering a cascade of inflammatory reactions and activating dendritic cells [4]. Bacterial infections from organisms like *Staphylococcus aureus* and *Streptococcus* produce large amounts of HAase, leading to significant HA degradation in the skin and causing cellulitis [5,6]. Numerous studies have demonstrated the potential anti-inflammatory effects of HAase inhibitors, including their ability to reduce the release of inflammatory factors [7,8,9]. According to current research progress, HAase inhibitors can be roughly divided into nine categories: alkaloids, antioxidants, terpenoids, flavonoids, synthetic compounds, mucopolysaccharide fatty acids, oligosaccharides, and anti-inflammatory drugs [10,11,12,13]. In recent years, reports on the inhibitory activity of natural product extracts on HAase have gradually increased [14,15]. Researchers recognize the significant potential for discovering enzyme inhibitors in natural products due to their diverse chemical structures and biological activities. Traditional methods for isolating target compounds from complex natural products include systematic separation and bioactivity-guided separation [16,17]. Currently, the specific enrichment of target components using solid-phase and micro-solid-phase extraction has become more common [18,19]. Magnetic nanoparticles (MNPs), known for their selective absorption and ease of synthesis, have garnered significant research attention [20,21,22]. Immobilizing enzymes on MNPs has proven effective for binding and isolating enzyme ligands in herbal extracts without destroying enzyme activity [23,24]. This method enables the rapid discovery of the active ingredients in herbal medicines.

Dan Shen, made from the dried roots of *Salvia miltiorrhiza* Bunge (Fam. Labiatae), is a popular traditional Chinese medicine. Its main chemical constituents include salvianolic acid B (SAB), Danshensu (DSS), cryptotanshinone, rosmarinic acid (RA), protocatechuic aldehyde, and tanshinone IIA, with SAB being the most abundant [25,26]. It is commonly used to treat conditions such as coronary artery disease, angina pectoris, myocardial infarction, cerebrovascular disease, various types of hepatitis, chronic renal failure, dysmenorrhea, etc. [27,28,29]. Furthermore, numerous studies have demonstrated its pharmacological bioactivities, including anti-atherosclerosis, anti-platelet aggregation, anti-inflammatory, and anti-oxidative effects [30]. However, there is limited research on the inhibition of hyaluronidase (HAase) activity by *S. miltiorrhiza* [31]. Some studies have found that members of the Labiatae family exhibit an inhibitory effect on HAase [32].

Although a great deal of work has been conducted to explore the protective effects of *S. miltiorrhiza* on the skin, studies on its role in combating skin inflammation through its inhibitory effect on hyaluronidase are still unclear, and the current screening of hyaluronidase inhibitors is still traditional and inefficient. This study aims to investigate HAase inhibitors in *S. miltiorrhiza* roots using magnetic ligand fishing technology. The HAase was first immobilized on magnetic nanomaterials, then the immobilized HAase was used to specifically adsorb compounds from the ethyl acetate fraction of *S. miltiorrhiza* root extract. Comparing the retention times of the obtained ligands in HPLC with the standards of the laboratory compounds revealed that the two ligands were SAB and RA, respectively. Subsequent in vitro enzyme activity assays confirmed their inhibitory effects on HAase. Notably, the inhibition of HAase by a specific ratio of SAB and RA (reflecting their proportion in the ethyl acetate fraction of the extract) was significantly higher than that of either compound alone. The median-effect equation and combination index indicated that the combination of SAB and RA strongly synergistically inhibited HAase at the specified ratios. Enzyme kinetics and molecular docking results showed that both SAB and RA act as mixed-type inhibitors of HAase, with SAB binding more easily to the enzyme. In addition, we observed that a 3:2 combination of SAB and RA significantly decreased the secretion of TNF-α, IL-1, and IL-6 inflammatory cytokines in UVB-irradiated HaCaT cells. In conclusion, combining these two compounds in the appropriate ratios holds significant potential for developing drugs to treat skin inflammation.

## 2. Results and Discussion

### 2.1. Characterization of Immobilized Hyaluronidase

#### 2.1.1. Transmission Electron Microscopy (TEM) Images

**Figure 1:**
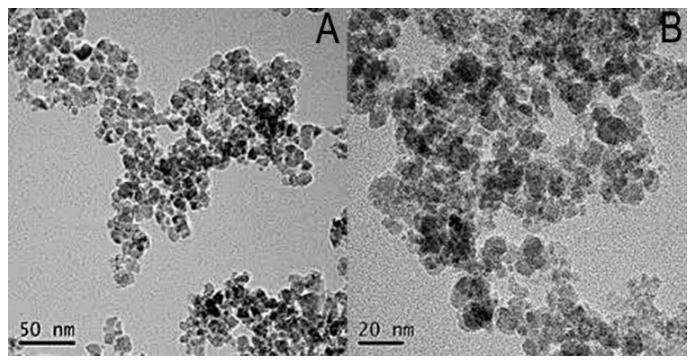
TEM images of MNPs@SiO_2_ (**A**) and MNPs@HAase (**B**).

The morphology and size of MNPs@SiO_2_ and MNPs@HAase were characterized by TEM, as shown in Figure 1. Both the MNPs@SiO_2_ and MNPs@HAase exhibited fine dispersibility, and their diameters ranged from 5 to 15 and from 10 to 20 nm, respectively. We found that having the enzyme immobilized on the magnetic nanomaterial did not impact the dispersibility of the material. Furthermore, the change in size of the material demonstrated the successful fixation of the enzyme.

#### 2.1.2. Fourier Transform Infrared (FT-IR) Spectra

**Figure 2:**
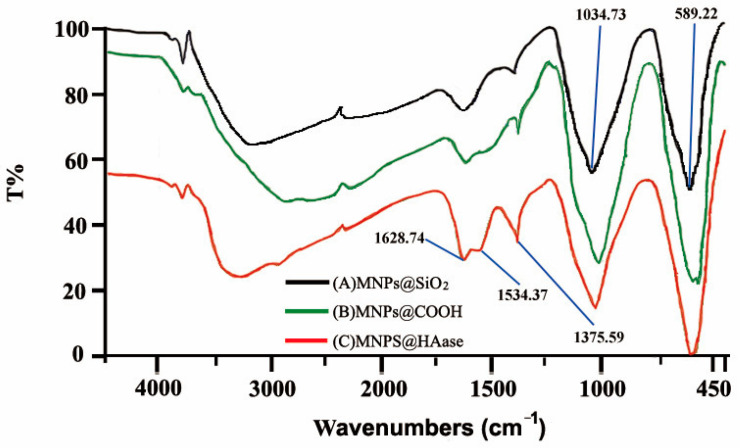
FT-IR spectra of MNPs@SiO_2_ (A), MNPs@COOH (B), and MNPs@Haase (C).

FT-IR confirmed the fabrication of MNPs@SiO_2_, MNPs@COOH and MNPs@HAase. Figure 2(A) shows a strong IR absorption band at 589 cm^−1^ that is characteristic of Fe-O bond stretching vibration, with another band at 1035 cm^−1^, indicating the asymmetric stretching vibration of the Si-O-Si bond, confirming the successful synthesis of MNPs@SiO_2_. In Figure 2(B), the IR absorption bands at 1035, 1534, and 1629 cm^−1^ correspond to the C-N bond bending vibrations, N-H bending vibrations, and C=O bond stretching vibrations, respectively. These peaks, attributable to amide bonds, indicate the successful bonding of MNPs@SiO_2_ with carboxyl groups. In addition, Figure 2(C) shows the higher intensity of the IR absorption peaks at 1035, 1534, and 1629 cm^−1^ compared to spectral line B, indicating the higher presence of carbonyl groups in MNPs@HAase and confirming successful enzyme immobilization on the magnetic materials.

#### 2.1.3. Magnetization Curves and Thermal Gravimetric Analysis Curves

**Figure 3:**
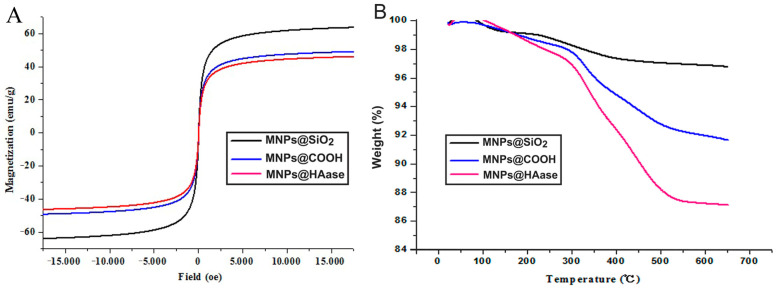
Magnetization curves (**A**) and TGA curves (**B**) of MNPs@SiO_2_, MNPs@COOH, and MNPs@HAase.

The magnetism of MNPs@SiO_2_, MNPs@COOH, and MNPs@HAase was characterized using a sample magnetometer (VSM) at room temperature, producing the magnetization curves shown in Figure 3A. All the magnetization curves passed through the origin, indicating that all three modified magnetic nanomaterials were super-magnetic, although their properties gradually weakened. The saturation magnetization values were 64.21 emu/g for MNPs@SiO_2_, 49.49 emu/g for MNPs@COOH, and 46.47 emu/g for MNPs@HAase. The decrease in magnetization intensity with the addition of carboxyl groups and HAase to the surface of MNPs@SiO_2_ further confirms the successful immobilization of HAase on the surface of the magnetic nanomaterials. 

The amount of HAase immobilized on MNPs was quantified by thermal gravimetric analysis (TGA). In the figure provided below, it is evident that MNPs@SiO_2_, MNPs@COOH, and MNPs@HAase exhibit similar weight loss patterns in two segments. Below 200 °C, all three nanomaterials experienced moisture loss (approximately 2% by weight) from their surfaces. Between 200 °C and 650 °C, the weight loss ratios varied among the three materials. The discrepancy in weight loss between MNPs@COOH and MNPs@HAase indicates the quantity of enzyme that has been immobilized on the surface of the carboxylated nanoparticles. Based on TGA, the relative enzyme binding capacity of the nanoparticles was calculated to be 45.28 µg/mg. VSM and TGA characterization indicated that HAase was successfully fixed to MNPs@COOH, and that the binding did not change the properties of the material.

### 2.2. Stability and Reusability of Immobilized Hyaluronidase

**Figure 4:**
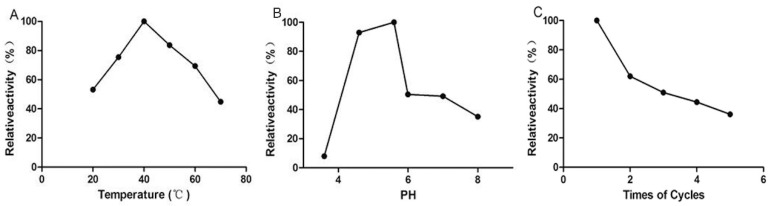
Effects of temperature (**A**) and pH (**B**) on the activity of MNPs@HAase and the reusability (**C**) of MNPs@HAase (the optimal activities for MNPs@HAase were taken as 100%).

The temperature and pH value are two important factors affecting the activity and stability of the immobilized enzyme. Firstly, different temperatures (20, 30, 40, 50, 60, and 70 °C) were tested under pH 5.6, revealing that 40 °C yielded the optimal results (Figure 4A). The results showed that the MNPs@HAase had good heat tolerance and maintained 45% of its activity at 70 °C. Secondly, the effect of different pH values (3.6, 4.6, 5.6, 6.0, 7.0, and 8.0) on MNPs@HAase was investigated at pH = 5.6, identifying pH 5.6 as optimal (Figure 4B). The MNPs@HAase retains more than 90% of its activity at pH 4.6. The enzyme’s reusability was assessed through repeated enzymatic reactions with MNPs@HAase, revealing that it maintained approximately 36% of its activity after six cycles (Figure 4C). To sum up, the immobilized enzyme shows good performance in terms of high temperature resistance, acid and alkali resistance, and reusability.

### 2.3. Ligand Fishing for Hyaluronidase in S. miltiorrhiza Roots

**Figure 5:**
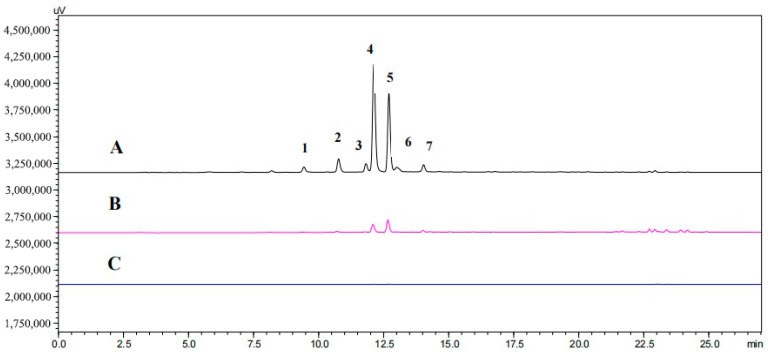
HPLC chromatogram of the ethyl acetate fraction of *Salvia miltiorrhiza* Bunge for S_0_ (A), S_5_ (B), and Blank S_5_ (C). (The numbers 1-7 in line A of the graph represent one peak, respectively.)

**Figure 6:**
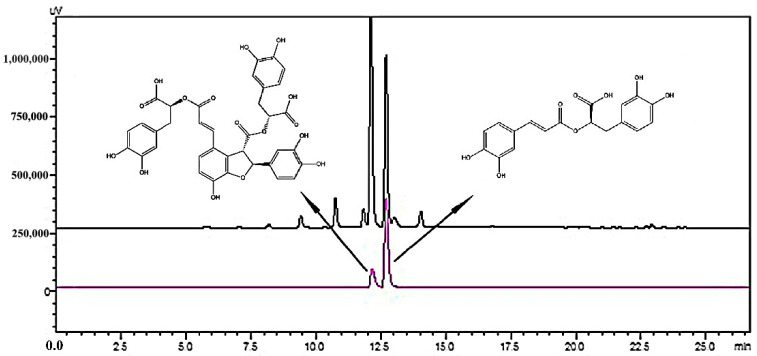
HPLC chromatogram of the ethyl acetate fraction of *S. miltiorrhiza* of S_0_ (black line), SAB, and RA (purple line). The chemical structure formulas of the two peaks of the purple line are shown by arrows.

The MNPs@HAase was used to extract enzyme ligands from the ethyl acetate fraction of *S. miltiorrhiza* root extract. Meanwhile, solid-phase extraction with MNPs@COOH was conducted as a blank control, resulting in the obtained S_5_ being named Blank S_5_. As shown in Figure 5, S_0_ displayed seven peaks, whereas S_5_ exhibited only two peaks. The absence of peaks in the combined MNP S_5_ indicated the specific adsorption of two peaks from the ethyl acetate fraction of *S. miltiorrhiza* root extracts by MNPs@HAase. By comparing the retention times of the two peaks of S_5_ to those of the main phenolic acids of *S. miltiorrhiza*, it was determined that peak 4 and peak 5 shared identical retention times with SAB and RA, respectively. As illustrated in Figure 6, HPLC analysis of the mixed standards of the two compounds revealed that they corresponded to peaks 4 and 5, confirming the identity of the two compounds as SAB and RA. The ligands that are specifically adsorbed by the immobilized enzyme can bind to the enzyme. Further testing is necessary to determine the influence of these ligands on HAase activity, in order to ascertain whether they act as HAase enzyme inhibitors or agonists.

### 2.4. Inhibitory Assays of Salvianolic Acid B and Rosmarinic Acid

The inhibition rates of the samples are presented in Table 1. The two HAase ligands, SAB and RA, were extracted from the ethyl acetate fraction of *S. miltiorrhiza* root extracts by MNPs@HAase, suggesting their potential as HAase inhibitors. To confirm whether these compounds inhibit HAase activity, an in vitro enzyme assay was employed, with ascorbic acid as the positive control. We observed that, under the same concentration conditions, SAB and RA exhibited a lower inhibitory effect on HAase compared to the EA of *S. miltiorrhiza.* It is our hypothesis that the two compounds present in the EA of *S. miltiorrhiza* may act synergistically to inhibit HAase. Meanwhile, we calculated the peak areas and ratios of SAB and RA in the ethyl acetate fraction of *S. miltiorrhiza* using the integration method. The inhibition of HAase activity by a mixture of these compounds in the following proportions (SAB:RA = 3:2) was tested. In order to determine whether the two compounds synergistically inhibit HAase, further studies were conducted. 

### 2.5. Data Analysis with the Combination Index Method 

**Figure 7:**
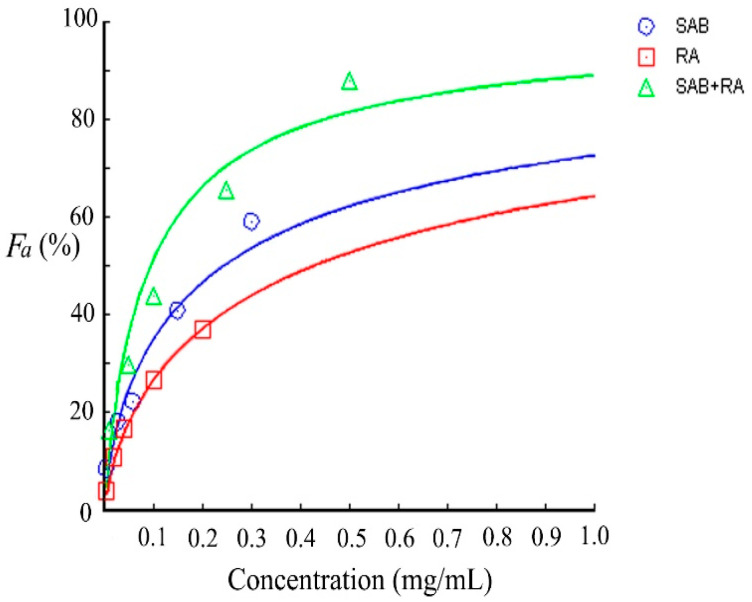
Dose-dependence of drug effect values *F_a_* for SAB (blue line) and RA (red line) used alone and for the combination of SAB and RA (green line); the results represent five independent experiments.

**Figure 8:**
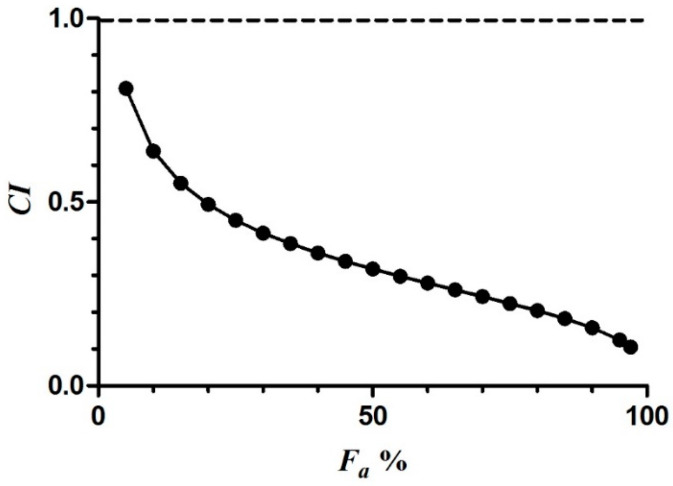
Compusyn V1.0 was used to synthesize *F_a_*-*CI* plots of the SAB and RA combinations, with each rounded point indicating a drug-combination-specific combinatorial index value CI. CI = 1 indicates a superimposed effect, CI > 1 indicates an antagonistic effect, and CI < 1 indicates a synergistic effect. (The dotted line is the threshold that distinguishes the mode of joint action of the compounds.)

As shown in Table 2 and Figure 7, when SAB and RA acted individually and in conjunction, their corresponding *F_a_* values tended to increase with the increase in drug concentration. Notably, the *F_a_* values of the combination group were higher than the *F_a_* values of the single-compound group. Table 3 presents the slopes of the neutralization curve equations (*m*), the median effective concentration (*D_m_*), and the correlation coefficients (*r*) for both drugs. Furthermore, *F_a_-CI* curves were plotted using *F_a_* values obtained from the simulation as horizontal coordinates and the corresponding *CI* values as vertical coordinates, as shown in Figure 8. Table 3 and Figure 8 reveal a positive correlation between the *F_a_* values and concentrations (*m* > 0, *r* > 0.9) in each group. Additionally, *D_m_* was smaller than the sum of the *D_m_* of the individual drugs. Notably, when *F_a_* > 5%, *CI* < 1, indicating synergistic effects from the combined action of the two drugs. Furthermore, as the *F_a_* values increased, the *CI* values gradually decreased, signifying the enhanced synergistic effects of the two drugs. We observed a significant dose-dependent inhibition of HAase by SAB and RA when used alone, as well as in combination. Furthermore, we found that HAase could even be synergistically inhibited at low concentrations when SAB and RA were combined.

### 2.6. Kinetic Studies of Ligands

**Figure 9:**
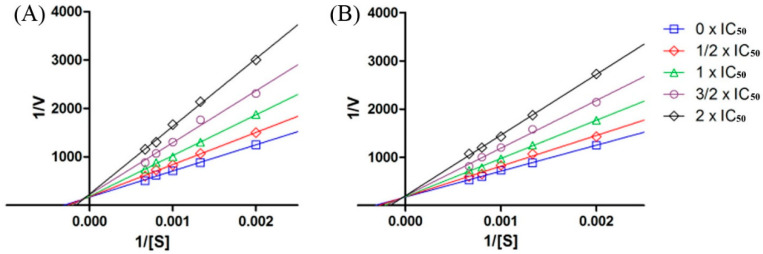
Lineweaver–Burk plots of HA enzyme inhibition by SAB (**A**) and RA (**B**) were obtained by plotting the inverse of the enzyme reaction rate at different IC_50_ conditions for SAB and RA (1/V) and the inverse of different substrate concentrations (1/[S]). Values are expressed as the mean ± SEM of triplicate experiments.

We constructed Lineweaver–Burk plots to investigate the mechanism of HAase inhibition by SAB and RA. Figure 9 shows that the Lineweaver–Burk plots for both SAB and RA are linear and intersect at a point in the second quadrant, suggesting that both SAB and RA are mixed-type inhibitors of HAase that compete with the substrate for binding sites on the enzyme. The K*_i_* value represents the relationship between the slope of the double inverse curve and the concentration of the inhibitor, while K*_is_* represents the relationship between the Y-intercept of the double inverse curve and the inhibitor concentration The K*_i_* and K*_is_* values were 0.22 and 0.96 µM for SAB, and 0.54 and 4.60 µM for RA, respectively. Notably, the K*_is_* values are more than four times higher than the K*_i_*_,_ value, suggesting that both compounds prefer to bind to HAase rather than to the enzyme-substrate complex.

### 2.7. Molecular Docking Studies of Ligands

**Figure 10:**
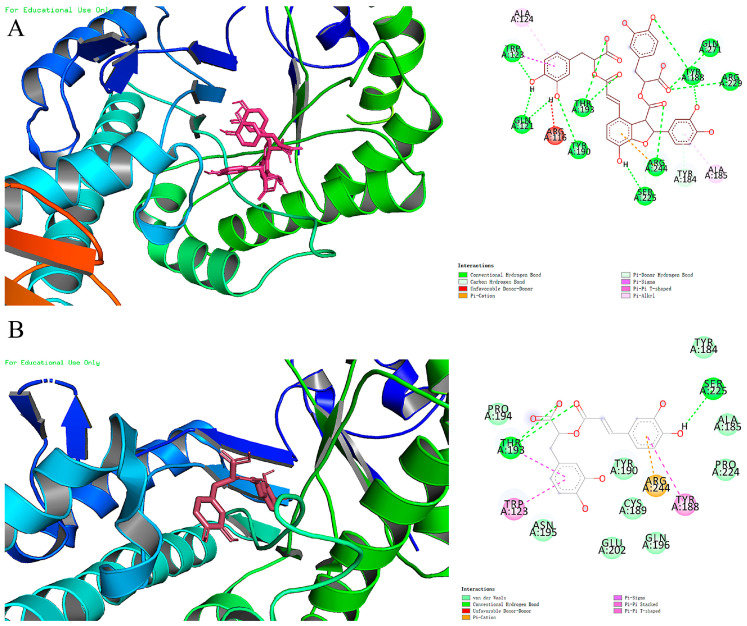
Molecular docking studies showing HAase with SAB (**A**) and RA (**B**).

The molecular docking results for SAB and RA with HAase showed that their lowest binding energies were −9.4 and −8.0 kcal/mol, respectively. SAB exhibited the lowest binding energy, indicating a higher affinity for HAase compared to RA. The 2D and 3D representations of the docking results (Figure 10) show that both compounds bind tightly to the active pocket of HAase. SAB formed stable hydrogen bonds with Gln-121, Gln-271, Thr-193, Tyr-190, Tyr-188, Ser-225, Trp-123, Arg-244, and Arg-229, and other amino acid residues. Additionally, Arg-244 formed *π*-cation interactions, Trp-123 engaged in π-σ interactions, and Tyr-184 formed carbon-hydrogen bonds with Ala-124 and Ala-185, contributing to π-alkyl interactions, and the formation of unstable hydrogen bonds in the figure are associated with Arg-116. RA, on the other hand, developed van der Waals forces with amino acid residues such as Pro-224, Pro-194, Asn-195, Tyr-184, Ala-185, Tyr-190, Asn-195, Glu-202, Gln-196, and Cys-189. It also established stable hydrogen bonds with Thr-193 and *π*-*σ* bonds, as well as *π*-*π* stacking interactions with Trp-123 and Tyr-188. 

### 2.8. Inhibition of Ligands on the Release of Inflammatory Cytokines in UVB-Injured HaCaT Cells

**Figure 11:**
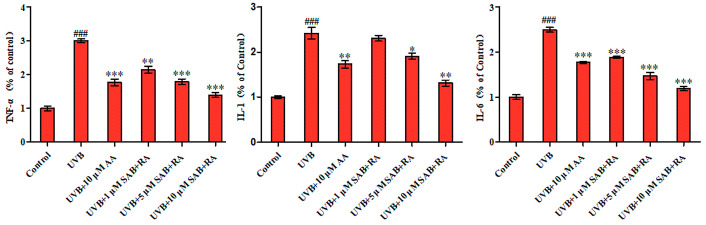
Effects of SAB/RA (3:2) on TNF-α, IL-6, and IL-1α levels in UVB-treated HaCaT cells. The cells were irradiated with UVB (200 mJ/cm2) and then treated with SAB/RA (3:2) at concentrations of 1, 5, and 10 μM, or AA at a concentration of 10 μM for 24 h. The values were presented as the mean ± SEM of triplicate experiments. ### *p*  <  0.001 indicates a significant difference compared to the control group; *** *p*  <  0.001, ** *p*  <  0.01, and * *p*  <  0.05 indicate significant differences compared to the UVB-treated group.

The ELISA results demonstrated that HaCaT cells were exposed to 200 mJ/cm^2^ UVB, leading to significantly higher secretion levels of the cytokines TNF-α, IL-1, and IL-6 compared to the control group. This indicates the successful establishment of the model. As depicted in Figure 11, the administration of a mixture of SAB and RA resulted in a significant reduction in the secretion of TNF-α, IL-1, and IL-6 in HaCaT cells that were irradiated by UVB when compared with the UVB group. These findings suggest that the combination of SAB and RA has an inhibitory effect on inflammatory cytokine secretion in UVB-exposed HaCaT cells. Given that a mixture of two HAase inhibitors, SAB and RA, has been shown to inhibit the secretion of inflammatory factors from skin cells, it could be inferred that they may both have an inhibitory effect on skin inflammation.

## 3. Materials and Methods

### 3.1. Materials

Samples weighing 5 kg were collected from Zhongjiang County, Sichuan Province, at longitude 104°26′–105°15′ E and latitude 30°31′–31°17′ N, and were identified as *S. miltiorrhiza* by Professor Yongmei Zhang of the Chengdu Institute of Biology, Chinese Academy of Sciences. A specimen was preserved at the Specimen Museum of the Chongqing Institute of Traditional Chinese Medicine, with the voucher number SM717306868, and the roots were sun-dried for later use. Hyaluronic acid sodium salt was purchased from Shanghai Macklin Biochemical and HAase was purchased from Shanghai Jizhi Biochemical (Shanghai, China). Acetylacetone (>99%) was purchased from Shanghai Macklin Biochemical (Shanghai, China); 4-(dimethylamino)-Benzaldehyde was purchased from Shanghai Macklin Biochemical (Shanghai, China); (3-Aminopropyl) trimethoxysilane (APTMS) and tetraethyl orthosilicate (TEOS) were purchased from TCI (Tokyo, Japan). HPLC-grade methanol was obtained from J&K Technology in Beijing, China, and HPLC-grade water was obtained from a water purification (18.25 MΩ) system (Ulupure, Chengdu, China). All other chemicals, solvents, and reagents were of analytical reagent grade or higher. DMEM medium, fetal bovine serum (FBS), and phosphate buffer solution (PBS) for cell culture were purchased from Biological Industries (Kibbutz Beit Haemek, Israel). TNF-α, IL-1, and IL-6 ELISA kits were purchased from Chongqing Tuo Shi Zhong He Biotechnology Co. (Chongqing, China). The acetic acid buffer solution had a concentration of 20 mM and a pH of 5.6.

### 3.2. Instruments and Equipment

The HPLC system includes a Shimadzu LC-20 AD series with a thermostatic column compartment, an SPD-20A UV-Vis detector, and a Kromasil 100-5-C18 column (4.6 × 250 mm). A PHS-3C pH meter from the Shanghai LeiCi Device Corporation was used to measure the pH value.

### 3.3. Preparation of Immobilized Hyaluronidase

#### 3.3.1. Preparation of Fe_3_O_4_ Magnetic Nanoparticles

Following examples in the previous literature, researchers synthesized MNPs using the co-precipitation method [21,22,33]. They dissolved 0.7455 g of FeCl_2_•4H_2_O and 2.0271 g of FeCl_3_•6H_2_O in 250 mL of deionized water under magnetic stirring, then added ammonia water until the pH values reached 9–10. After a 0.5-h reaction, a magnet separated the product, which was then washed three times with alternating ethanol and deionized water.

#### 3.3.2. Preparation of MNPs@SiO_2_ Magnetic Nanoparticles

The Fe_3_O_4_ obtained in the previous step was dispersed into 150 mL ethanol by ultrasonic treatment for 1 min. Then, we added 400 µL of TEOS to the reaction system and ammonia water until the pH reached 9–10. After a 5-h reaction at 35 °C, a magnet separated the product, which was washed three times with alternating ethanol and deionized water.

#### 3.3.3. Preparation of MNPs@NH2 Magnetic Nanoparticles

The MNPs@SiO_2_ obtained from the previous step were dispersed into 90 mL ethanol containing 0.75 mL of deionized water. Then, 2 mL of APTMS was added to the product. After 24 h of reaction at 35 °C, the product was separated by a magnet and washed three times with alternating ethanol and deionized water. The product was freeze-dried for backup.

#### 3.3.4. Preparation of MNPs@COOH Magnetic Nanoparticles

First, 500 mg of freeze-dried MNPs@NH_2_ and 3 g of succinic anhydride were added to 30 mL of DMF using the ultrasonic method for 1 min. After a 3-h reaction period, the same treatment was given as described in Section 3.3.3.

#### 3.3.5. Immobilization of Hyaluronidase

The researchers activated MNPs@COOH with MES buffer (50 mM, pH 6.5, 10 mM EDC, and 20 mM NHS) for 0.5 h. They covalently bound HAase to the MNPs through overnight incubation [34]. Magnetic separation and repeated washing removed the free enzyme, resulting in MNPs@HAase. The three types of MNPs composites, i.e., MNPs@SiO_2_, MNPs@COOH, and MNPs@HAase, were characterized by TEM, FT-IR, VSM, and TGA.

### 3.4. Ligand Fishing from Salvia miltiorrhiza Root Extract

The roots of *S. miltiorrhiza* (10 g) were powdered and extracted with 100 mL of 80% ethanol at 80 °C for 1 h. After evaporating the ethanol, we extracted the remaining aqueous solution with ethyl acetate. The supernatant was then filtered and concentrated to dryness using a rotary evaporator. Before performing ligand fishing, the extracts were prepared at a concentration of 1 mg/mL with acetic acid buffer solution, which was named S_0_. We incubated 1 mL of S_0_ with 20 mg of MNPs@HAase in a 5 mL centrifuge tube, stirring with a vortex oscillator at 37 °C for 30 min. The MNPs@HAase was then separated from the solution with a magnet and washed 3 times with an acetic acid buffer solution. To desorb the ligands bound to MNPs@HAase, we used 0.5 mL of 50% acetonitrile. The eluate was collected and labeled S_5_. 

To identify the ligands, we analyzed S_0_ and S_5_ using HPLC. The column temperature was set to 35 °C, with UV detection at a wavelength of 254 nm. The mobile phase consisted of solvent A (0.1% formic acid in water) and solvent B (methanol), using a gradient elution from 30% to 100% B over 30 min. The flow rate was 0.8 mL/min. 

### 3.5. Inhibitory Assay

The inhibition assay of the ligands against HAase was performed, following the Elson–Morgan modified method, and the working concentration of the sample was determined to be 0.5 mg/mL [35,36,37]. The assays took place in 10 mL centrifuge tubes. Firstly, 0.5 mL of sample solution and 0.5 mL of HAase were added (500 U/mL) to the tube in a 37 °C water bath for 20 min. Then, 0.1 mL of CaCl_2_ (2.5 mol/L) solution was added to the tube in a 37 °C water bath for 20 min. Next, 0.5 mL of sodium hyaluronate solution (0.5 mg/mL) was added to the tube at 37 °C in a water bath for 40 min. Afterward, we added 0.5 mL of distilled water, 0.1 mL of NaOH solution, and 0.5 mL of acetylacetone (composed of 50 mL of Na_2_CO_3_ (1 M) and 3.5 mL of acetylacetone) to the tube. This mixture was kept in the 37 °C water bath for 15 min and then transferred to an ice bath for 10 min, followed by 10 min at room temperature. Afterward, 1 mL of P-DAB and 4.3 mL of ethanol were added to it. The absorbance was measured at 530 nm after allowing the solution to stand for 30 min. The HAase inhibition rate (*R*) was calculated using the following formula:

(1) $$R=\left[\left(A_{0}-A\right)/A_{0}\right]\times 100\%$$

where *A*_0_ is the absorbance of the control group (without the sample, enzyme solution, and sodium hyaluronate as the drug control), and *A* is the absorbance of the sample group (with the sample, no enzyme solution, and sodium hyaluronate as the blank control); ascorbic acid was the positive control.

### 3.6. Evaluation of Combined Effects of Salvianolic Acid B and Rosmarinic Acid 

The *F_a_* value for each group is *F_a_* (%) = R* (where R* denotes the mean value of inhibition in the administered group). Neutralization graphs (y = log *F_a_*/*F_u_*, x = log*D*, and y = ax + b) and the neutralization of the two components in monotherapy and combination were calculated based on the neutralization equation *F_a_*/*F_u_* = (*D*/*D_m_*)*^m^* [38]. In this equation, *F_a_* is the drug effect, *F_u_* = 1 − *F_a_*, *D* is the drug concentration, *D_m_* is the neutralization concentration, that is, the concentration of the drug when it exerts 50% of its effect, and m is the slope of the equation of the neutralization curve. Dose was calculated using (log*D_m_* = −b/a, a = m) and the corresponding parameters of the equation (slope *m* and correlation coefficient *r*, etc.). Then, the *CI* value of the combined effect was calculated according to the following formula:

(2) $$CI=\frac{D_{1}}{D_{x1}}+\frac{D_{2}}{D_{x2}}$$

where *D*_1_ and *D*_2_ are the concentrations of each of the two drugs causing the x effect when combined, and *D_x_*_1_ and *D_x_*_2_ are the concentrations of each of the two drugs causing the x effect when used alone. A *CI* value of 1 indicates an additive effect, while a *CI* > 1 suggests antagonism, and a *CI* < 1 indicates synergy [39]. The quantitative effect-relationship curves of the two drugs individually and after combination were plotted using GraphPad Prism V5.0, with the drug concentration as the horizontal coordinate and the *F_a_* value as the vertical coordinate. The data of the combined action group were simulated using Compusyn V1.0 and the neutral effect equation. The *F_a_-CI* curves were plotted using GraphPad Prism V5.0, with the simulated *F_a_* on the horizontal axis and its corresponding *CI* value on the vertical axis. This comprehensive analysis aimed to thoroughly evaluate the effects of the 2 drugs following their combined action, based on the provided data.

### 3.7. Enzyme Kinetics Study

We conducted enzyme kinetic studies to explore the inhibitory patterns of the ligands of HAase, employing a method that was previously documented [40]. Based on the earlier HAase inhibition assay, 5 different concentrations of ligands (0, 1/2 × IC_50_, 1 × IC_50_, 3/2 × IC_50_, and 2 × IC_50_) were reacted with 5 different concentrations of sodium hyaluronate (500, 750, 1000, 1250, and 1500 µM). The reaction rate was monitored within the initial 30 min post-initiation. Grouping the samples by concentration, we derived the corresponding regression equations to construct the Lineweaver–Burk diagram, with the reciprocal of the average reaction rate (1/V) on the ordinate and the reciprocal of the substrate concentration (1/[S]) on the abscissa. From this diagram, we extracted the reciprocal of the transverse intercept (K*_m_*) and longitudinal intercept (Vmax) to compute the values of the constants K*_i_* and K*_is_*.

### 3.8. Molecular Docking Study

Molecular docking studies were performed using Autodock Vina 1.1.2, while the results were analyzed and the images processed using Pymol 2.1 and Discovery Studio 3.0 [41]. Firstly, the 2D structures of rosmarinic acid and salvianolic acid B were downloaded from the compound database (https://pubchem.ncbi.nlm.nih.gov/, accessed on 15 March 2024), and the structures were optimized using the Chembio 3D Ultra 14.0. Then, the 3D structures of HAase enzyme proteins were obtained by downloading them from the Protein Structure Data Bank (PDB Code: 2J88, http://www.rcsb.org/pdb/home/home.do, accessed on 16 January 2024). Next, the Autodock Tools 1.5.6 software was used to set the ligand and receptor parameters and convert their format to PDBQT for subsequent use. Finally, Autodock Vina 1.1.2 facilitated the generation and storage of molecular docking outcomes and enabled their visual analysis.

### 3.9. Inhibition of TNF-α, IL-1 and IL-6 Production Assay in HaCaT Cells

#### 3.9.1. Cell Culture

HaCaT cells were inoculated in cell culture dishes with DMEM medium containing 10% fetal bovine serum (FBS) and 1% streptomycin-penicillin, cultured in an incubator at 37 °C and 5% CO_2_. When the cell adhesion reached 70%~80% and showed a logarithmic period, the adherent HaCaT cells were digested with trypsin and added into DMEM complete medium, then gently blasted with a pipette gun to completely shed the cells. The cell suspension was collected and centrifuged in a centrifuge tube at 1000× *g* for 5 min, then the supernatant was discarded, 1 mL of culture medium was added, the cells were blown away with a pipette gun, and the cells were placed in an incubator for subculture.

#### 3.9.2. Effects of Ligands on the Release of TNF-α, IL-1, and IL-6

HaCaT cell precipitates were collected during the logarithmic growth stage, and the cell density was adjusted to 1 × 10^5^ cells/mL. Subsequently, 2 mL of cell suspension was added to each well of a 6-well cell culture plate. The plate was then incubated in a 37 °C incubator with 5%CO_2_. Once the cells reached more than 80% adhesion and entered the logarithmic growth phase, the cell culture medium for each group was removed, it was washed once with PBS, and a small amount of PBS was added to cover the underside. Aluminum foil paper was used to protect the cells from light, and then the designated wells were opened for UVB irradiation at an intensity of 200 mJ/cm^2^ [42]. The experimental groups were divided into the control group, UVB group, and dosed group. In the dosed group, different concentrations (1 μM, 5 μM, and 10 μM) of SAB/RA (3:2) were added after irradiation. After 24 h of incubation, supernatant culture solution samples were taken and analyzed using an ELISA kit to detect their effects on TNF-α, IL-1, and IL-6 release. Ascorbic acid (AA) served as a positive control.

## 4. Conclusions

Screening the HAase inhibitors found in traditional Chinese medicine extracts plays a crucial role in developing new drugs or new products for treating skin inflammation. This work established a rapid screening method for components with potential anti-inflammatory activity by immobilizing HAase on magnetic nanoparticles. We screened SAB and RA from *S. miltiorrhiza*, finding that both had similar inhibitory effects on HAase. When SAB and RA were combined in a fixed ratio of 3:2, their inhibitory effects on HAase demonstrated a synergistic effect, which increased with higher concentration. In addition, the combination significantly reduced the secretion of TNF-α, IL-1, and IL-6 inflammatory cytokines in UVB-irradiated HaCaT cells. Therefore, SAB and RA are important in developing treatments for skin inflammation.

## Figures and Tables

**Table 1:** Inhibitory effects of the samples against HAase.

Samples ^a^	Inhibition Rate (%) ^b^	IC_50_ (µg/mL)
*S. miltiorrhiza*	35.42 ± 2.24	1057.47 ± 64.45
EA of *S. miltiorrhiza*	80.86 ± 0.74	158.77 ± 4.57
SAB	68.27 ± 0.71	204.15 ± 1.08
RA	53.93 ± 2.21	450.24 ± 2.31
SAB/RA = 3:2	88.04 ± 0.71	112.83 ± 4.11
Ascorbic acid	28.43 ± 0.13	780.00 ± 3.62

^a^ Sample concentration (0.5 mg/mL), ^b^ values are expressed as the mean ± SD (*n* = 3).

**Table 2:** SAB-only group, RA-only group, and combined group *F_a_* values.

Groups	Concentration (mg/mL)	*F_a_* (%)
SAB	0.006	8.30
	0.03	18.07
	0.06	22.16
	0.15	40.98
	0.3	59.08
RA	0.004	3.80
	0.02	10.75
	0.04	16.83
	0.1	26.83
	0.2	36.99
SAB + RA	0.006 + 0.004	16.40
	0.03 + 0.02	29.80
	0.06 + 0.04	43.90
	0.15 + 0.1	65.60
	0.3 + 0.2	88.04

**Table 3:** Calculation results of *m*, *D_m_*, and *r* for the SAB-only group, RA-only group, and combined group.

Groups	*m*	*D_m_* (mg/mL)	*r*
SAB	0.69279	0.24238	0.98216
RA	0.69042	0.42646	0.99935
SAB + RA	0.88718	0.09295	0.95988

## Data Availability

The authors declare that all the data supporting the findings of this study are contained within the paper.
